# Immunotherapy combined with antiangiogenic therapy as third‐ or further‐line therapy for stage IV non‐small cell lung cancer patients with ECOG performance status 2: A retrospective study

**DOI:** 10.1002/cam4.7349

**Published:** 2024-06-13

**Authors:** Shuo Li, Ze‐Shun Yu, Hong‐Zhi Liu, Shu‐Jing Li, Ming‐Yue Wang, Fang‐Ling Ning, Li‐Jun Tian

**Affiliations:** ^1^ Department of Oncology Binzhou Medical University Hospital Binzhou Shandong People's Republic of China; ^2^ Department of Orthopedics Binzhou Medical University Hospital Binzhou Shandong People's Republic of China

**Keywords:** antiangiogenic therapy, Eastern Cooperative Oncology Group, immunotherapy, non‐small cell lung cancer

## Abstract

**Background:**

Patients with Eastern Cooperative Oncology Group performance status (ECOG PS) 2 probably cannot tolerate chemotherapy or other antitumor therapies. Some studies have reported that immunotherapy combined with antiangiogenic therapy is well‐tolerated and shows good antitumor activity. However, the efficacy of this combination as a later‐line therapy in patients with ECOG PS 2 is unclear. This study evaluated the effectiveness and safety of this combination strategy as third‐ or further‐line therapy in stage IV non‐small cell lung cancer (NSCLC) patients with ECOG PS 2.

**Methods:**

In this retrospective study, patients treated with camrelizumab plus antiangiogenic therapy (bevacizumab, anlotinib, or recombinant human endostatin) were included. Objective response rate (ORR), disease control rate (DCR), progression‐free survival (PFS), overall survival (OS), quality of life (QOL) assessed by ECOG PS, and safety were analyzed.

**Results:**

Between January 10, 2019, and February 28, 2024, a total of 59 patients were included. The ORR was 35.6% (21/59) and the DCR was 86.4%. With a median follow‐up of 10.5 months (range: 0.7–23.7), the median PFS was 5.5 months (95% confidence interval [CI]: 3.8–7.3) and the median OS was 10.5 months (95% CI: 11.2–13.6). QOL was improved (≥1 reduction in ECOG PS) in 39 patients (66.1%). The most common Grade 3–4 treatment‐related adverse events were hepatic dysfunction (6 [10%]), hypertension (5 [8%]), and hypothyroidism (3 [5%]). There were no treatment‐related deaths.

**Conclusions:**

Third‐ or further‐line immunotherapy combined with antiangiogenic therapy is well‐tolerated and shows good antitumor activity in stage IV NSCLC patients with ECOG PS 2. Future large‐scale prospective studies are required to confirm the clinical benefits of this combination therapy.

## 1 INTRODUCTION

Lung cancer has high incidence and mortality rates, accounting for 20% of global cancer‐related deaths.^1^ Non‐small cell lung cancer (NSCLC) accounts for more than 80% of lung cancer cases and is the most common histological type of lung cancer.^2^, ^3^ Due to the high aggressiveness of NSCLC and the lack of an effective early screening approach, 68% of lung cancer patients in China have stage IV disease at diagnosis.^4^ In the real world, a high proportion of patients has an Eastern Cooperative Oncology Group performance status (ECOG PS) of 2, characterized by multiple previous antitumor therapies.^5^ These patients typically have poor physical conditions and cannot tolerate chemotherapy or other antitumor therapies. Palliative treatment and supportive care are needed for these patients. Nevertheless, most of them and their families are willing to keep trying antitumor therapy.

Immune checkpoint inhibitors (ICIs), such as programmed death‐1 (PD‐1) inhibitors, programmed death‐ligand 1 (PD‐L1) inhibitors, and cytotoxic T lymphocyte‐associated antigen‐4 (CTLA‐4) inhibitors, provide a new alternative option for patients with stage IV NSCLC, especially for those without driver gene mutations.^6^ PD‐1/PD‐L1 inhibitor monotherapy has been approved by the Food and Drug Administration (FDA) for the treatment of patients with high PD‐L1 expression.^7^, ^8^, ^9^ Based on relevant clinical trials, ICIs have shown long‐term clinical benefits in patients with high PD‐L1 expression and ECOG PS 0–1.^10^, ^11^, ^12^ However, for patients with ECOG PS 2, data on the treatment of ICIs are limited.^13^ In addition, due to the high cost of testing and the spatiotemporal heterogeneity of PD‐L1 expression, as well as challenges associated with immunohistochemical detection,^14^ the expression of PD‐L1 is unknown in most patients. Therefore, investigating combination therapies to enhance the efficacy of anti‐PD‐1/PD‐L1 therapy has great clinical significance.^15^, ^16^

Abnormalities in the structure and function of tumor blood vessels lead to tumor growth and drug resistance. Antiangiogenic therapy can normalize tumor blood vessels and improve treatment outcomes. Despite the promising results in NSCLC, the use of antiangiogenic therapy as part of combination therapy is unsatisfactory in the first‐ or second‐line setting, and most clinical trials of antiangiogenic small‐molecule targeted drugs have failed. In this context, the ALTER0303 trial showed that third‐line treatment with anlotinib (a multitarget small‐molecule antiangiogenic drug) improved progression‐free survival (PFS) in NSCLC patients.^17^ However, this treatment mainly focused on patients with ECOG PS 0–1. At present, no targeted treatment regimens have been developed for patients with ECOG PS 2, which highlights the need for new treatment options.

Immunotherapy combined with antiangiogenic therapy has emerged in recent years. Preclinical studies have shown the potential synergistic effect of this combination.^18^, ^19^, ^20^ Antiangiogenic drugs can not only reverse the immunosuppressive effect caused by vascular endothelial growth factor (VEGF), but also normalize the tumor vascular system and promote the delivery of T cells and other immune effector molecules.^21^, ^22^, ^23^ Moreover, ICIs can normalize the tumor vascular system and increase the infiltration and killing function of effector T cells by activating them. Immune revitalization and normalization of tumor vasculature can ultimately lead to immune‐mediated tumor eradication.^24^

Relevant clinical trials have been conducted to evaluate the clinical benefits of immunotherapy combined with antiangiogenic therapy for stage IV NSCLC. ICI in combination with anlotinib was well‐tolerated and showed clinical efficacy as first‐line treatment for patients with stage IV NSCLC.^19^, ^25^ The phase 2 WJOG10718L study showed a promising objective response rate (ORR) and disease control rate (DCR) with first‐line atezolizumab plus bevacizumab in patients with non‐squamous NSCLC.^26^ Nevertheless, the efficacy and safety of this combination strategy as later‐line therapy in patients with ECOG PS 2 remain unclear. Therefore, this retrospective study investigated the effectiveness and safety of immunotherapy combined with antiangiogenic therapy as third‐ or further‐line therapy in stage IV NSCLC patients with ECOG PS 2. Furthermore, the impact of this combination therapy on quality of life (QOL) was also explored.

## 2 MATERIALS AND METHODS

### 2.1 Study design and patients

In this retrospective study, stage IV NSCLC patients with ECOG PS 2 who were treated with third‐ or further‐line immunotherapy combined with antiangiogenic therapy were included. Patients were excluded if they had previously received immunotherapy or antiangiogenic therapy before the study treatment. Data were obtained from the patients' electronic medical records. Disease staging was determined by the 8th edition of the American Joint Committee on Cancer (AJCC) staging system. The study was conducted in accordance with the principles of the Declaration of Helsinki and was approved by the Research Ethics Committee of Binzhou Medical University Hospital. Written informed consent was obtained from each individual.

### 2.2 Treatment

The drug used as immunotherapy was camrelizumab at a dose of 200 mg once every 3 weeks. Antiangiogenic drugs included bevacizumab (7.5 mg/kg once every 3 weeks), anlotinib (8–12 mg on days 1–14 of each 21‐day cycle), and recombinant human endostatin (210 mg once daily in each 21‐day cycle).

### 2.3 Follow‐up and assessments

Tumor response was assessed according to the Response Evaluation Criteria in Solid Tumors (RECIST), version 1.1. Chest‐enhanced computed tomography was performed every 6 weeks.

ECOG PS was assessed 1 day before treatment and every month after treatment. ECOG PS was scored as follows: 0 point indicates normal activity; 1 point indicates that patients are restricted in physically strenuous activity but ambulatory; 2 points indicate that patients can move freely for more than half the day but are unable to carry out any work activity; 3 points indicate that patients are confined to bed more than half the day; 4 points indicate that patients cannot take care of themselves; 5 points indicate death.^27^

Treatment‐related adverse events (TRAEs) were assessed and graded according to the National Cancer Institute Common Terminology Criteria for Adverse Events, version 5.0.

### 2.4 Outcomes

Outcomes included ORR, DCR, PFS, overall survival (OS), QOL, and TRAEs. ORR was defined as the proportion of patients with complete response (CR) or partial response (PR). DCR was defined as the proportion of patients with CR, PR, or stable disease (SD). PFS was defined as the time interval from the beginning of treatment to the occurrence of disease progression or death. OS was defined as the time from the beginning of treatment to death. Change in ECOG PS was used to evaluate the patients' QOL. Improved QOL was defined as ≥1 reduction in ECOG PS, whereas worsened QOL was defined as ≥1 increase in ECOG PS.

### 2.5 Statistical analysis

Statistical analyses were performed using SPSS 24.0 (IBM, Armonk, NY, USA) and GraphPad Prism 9.00 (GraphPad Software, San Diego, CA, USA). PFS and OS were estimated using the Kaplan–Meier method and the comparisons of survival between subgroups were performed using the log‐rank test. Univariable and multivariable Cox proportional hazard models were used to analyze the associated factors of survival. *p*‐value <0.05 was considered statistically significant.

## 3 RESULTS

### 3.1 Clinical characteristics and treatment strategies

Between January 10, 2019, and February 28, 2024, a total of 59 patients were included in this study. The baseline demographic and clinical characteristics are shown in Table 1. The median age was 67 years (range, 38–90), and 34 patients (57.6%) were over 65 years. All patients had no driver gene mutations. The histological subtypes were squamous cell carcinoma in 22 (37.3%) patients and adenocarcinoma in 37 (62.7%) patients. Eleven (18.6%) patients had brain metastasis and 10 (16.9%) had liver metastasis.

**TABLE 1:** Baseline characteristics of the study population.

Characteristics	Patients (*n* = 59)
Gender, *n* (%)	
Male	41 (69.5)
Female	18 (30.5)
Age group, *n* (%)	
<65 years	25 (42.4)
≥65 years	34 (57.6)
ECOG PS, *n* (%)	
2	59 (100)
Histology, *n* (%)	
Adenocarcinoma	37 (62.7)
Squamous cell carcinoma	22 (37.3)
Smoking history, *n* (%)	
Ever	45 (76.3)
Never	14 (23.7)
Treatment line, *n* (%)	
3	14 (23.7)
≥4	45 (76.3)
Brain metastasis, *n* (%)	
No	48 (81.4)
Yes	11 (18.6)
Liver metastasis, *n* (%)	
No	49 (83.1)
Yes	10 (16.9)
Immunotherapy, *n* (%)	
Camrelizumab	59 (100)
Antiangiogenic therapy, *n* (%)	
Anlotinib	32 (54.2)
Recombinant human endostatin	20 (33.9)
Bevacizumab	7 (11.9)
PD‐L1 status, *n* (%)	
Positive (TPS ≥1%)	2 (3.4)
Negative (TPS <1%)	2 (3.4)
Unknown	55 (93.2)

Abbreviations: ECOG PS, Eastern Cooperative Oncology Group performance status; PD‐L1, programmed death‐ligand 1; TPS, tumor proportion score.

### 3.2 Adverse events

TRAEs that occurred during the treatment period are shown in Table 2. The overall incidence of TRAEs was 86% (51/59), and Grade 3–4 TRAEs were reported in 25% of patients (15/59). The most common Grade 3–4 TRAEs were hepatic dysfunction (6 [10%]), hypertension (5 [8%]), and hypothyroidism (3 [5%]). No patients discontinued treatment due to TRAEs, and there were no treatment‐related deaths or life‐threatening adverse events. No new or unexpected TRAEs were identified in this study.

**TABLE 2:** Treatment‐related adverse events.

Event, *n* (%)	Patients (*n* = 59)		
	Any grade	Grade 1–2	Grade 3–4
Fatigue	13 (22)	13 (22)	0
Diarrhea	10 (17)	9 (15)	1 (2)
Nausea/vomiting	11 (19)	11 (19)	0
Pruritus	9 (15)	9 (15)	0
Hypertension	15 (25)	10 (17)	5 (8)
Hand‐foot syndrome	9 (15)	8 (14)	1 (2)
Oral mucositis	2 (3)	2 (3)	0
Rash	10 (17)	9 (15)	1 (2)
Hepatic dysfunction	14 (24)	8 (14)	6 (10)
Renal dysfunction	7 (12)	7 (12)	0
Proteinuria	11 (19)	11 (19)	0
Hypothyroidism	8 (14)	5 (9)	3 (5)
Platelet count decreased	7 (12)	6 (10)	1 (2)
Dizziness	11 (19)	10 (17)	1 (2)
White blood cell decreased	10 (17)	9 (15)	1 (2)
Anemia	4 (7)	4 (7)	0
Interstitial pneumonia	5 (9)	4 (7)	1 (2)
Enteritis	1 (2)	1 (2)	0

### 3.3 Treatment outcomes

**FIGURE 1:**
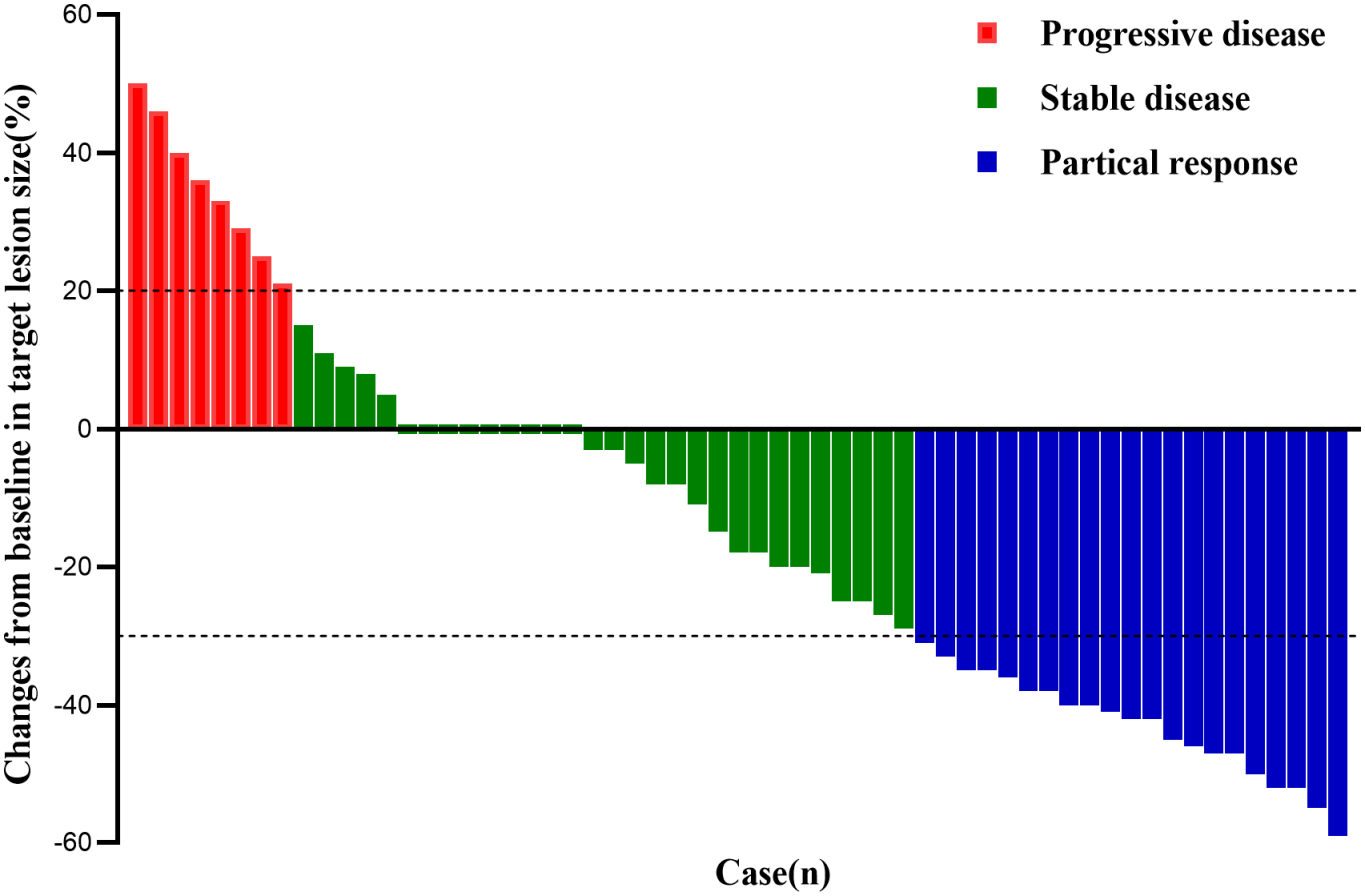
Waterfall plot illustrating maximum change in target lesion size from baseline (*n* = 59).

**FIGURE 2:**
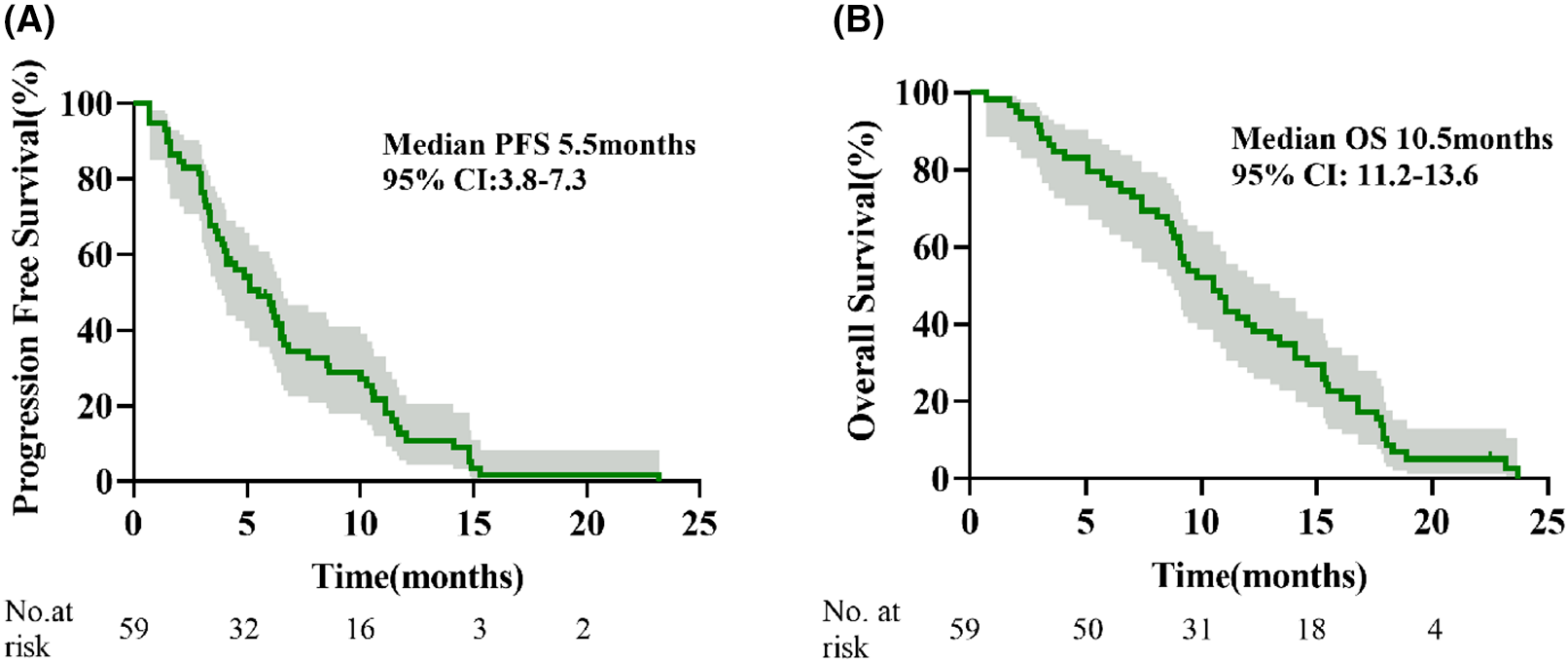
Kaplan–Meier survival curves. (A) Progression‐free survival. (B) Overall survival. CI, confidence interval.

**FIGURE 3:**
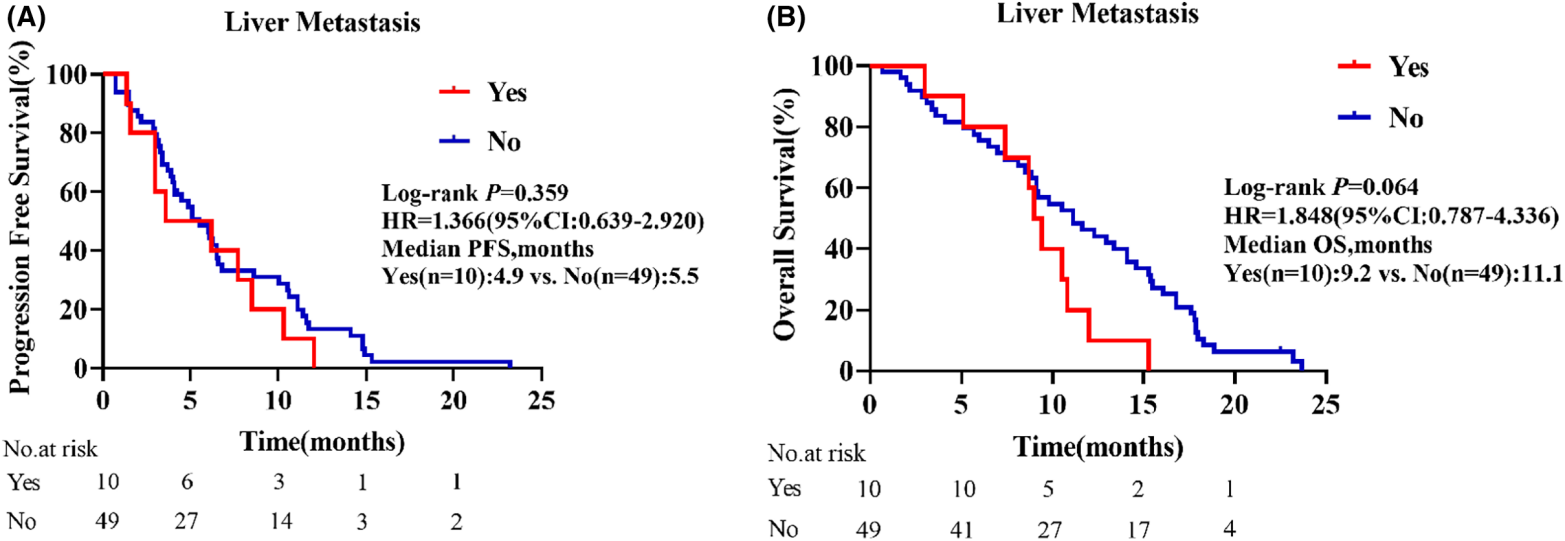
Survival outcomes in subgroups by the presence or absence of liver metastasis. (A) Progression‐free survival. (B) Overall survival. CI, confidence interval; HR, hazard ratio.

**FIGURE 4:**
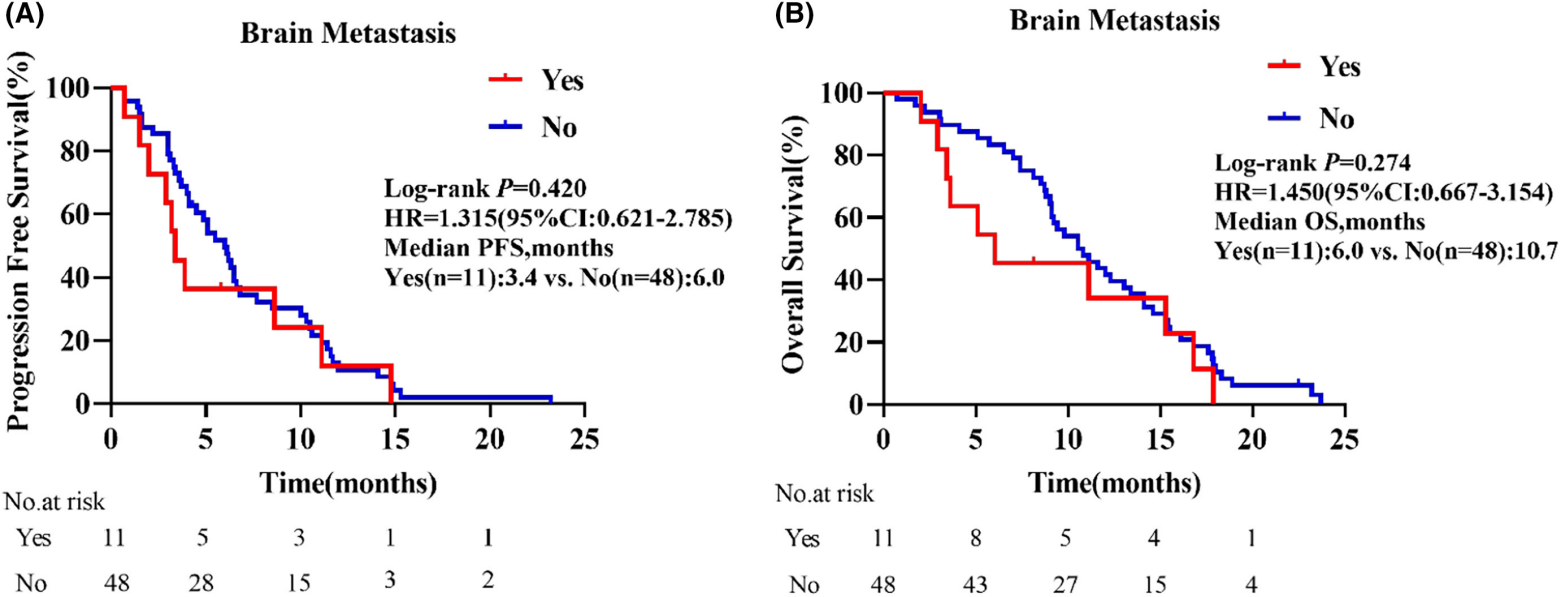
Survival outcomes in subgroups by the presence or absence of brain metastasis. (A) Progression‐free survival. (B) Overall survival. CI, confidence interval; HR, hazard ratio.

As displayed in the waterfall plot (Figure 1), the ORR was 35.6% (21/59), and the DCR was 86.4% (51/59). By the data cutoff date on February 28, 2024, the median follow‐up duration was 10.5 months (range, 0.7–23.7). Median PFS was 5.5 months (95% confidence interval [CI]: 3.8–7.3) and the median OS was 10.5 months (95% CI: 11.2–13.6; Figure 2). In the subgroup analysis, patients with liver metastasis had shorter median PFS (4.9 vs. 5.5 months, hazard ratio [HR] = 1.366, 95% CI: 0.639–2.920, *p* = 0.359) and OS (9.2 vs. 11.1 months, HR = 1.848, 95% CI: 0.787–4.336, *p* = 0.064) than those without liver metastasis, but the differences were not statistically significant (Figure 3). Compared with patients without brain metastasis, patients with brain metastasis had shorter median PFS (3.4 vs. 6.0 months, HR = 1.315, 95% CI: 0.621–2.785, *p* = 0.420) and OS (6.0 vs. 10.7 months, HR = 1.450, 95% CI: 0.667–3.154, *p* = 0.274), but no statistically significant differences were observed (Figure 4).

The univariable Cox regression analysis showed that smoking history and treatment line were potentially associated with PFS (Table 3) and OS (Table 4). Multivariable analysis revealed that the presence of smoking history was independently associated with worse PFS (HR = 2.379, 95% CI: 1.224–4.625, *p* = 0.011; Table 3) and OS (HR = 2.491, 95% CI: 1.244–4.988, *p* = 0.010; Table 4). Age, gender, histology, liver metastasis, brain metastasis, and type of antiangiogenic drug had no impact on PFS or OS.

**TABLE 3:** Univariable and multivariable Cox regression analyses of factors associated with progression‐free survival.

Characteristics	Univariable analysis			Multivariable analysis		
	HR	95% CI	*p*‐value	HR	95% CI	*p*‐value
Age (<65 vs. ≥65 years)	0.980	0.576–1.667	0.940			
Gender (female vs. male)	1.437	0.789–2.618	0.236			
Smoking history (ever vs. never)	2.590	1.344–4.993	0.004	2.379	1.224–4.625	0.011
Histology (adenocarcinoma vs. SCC)	1.268	0.737–2.182	0.391			
Brain metastasis (yes vs. no)	1.321	0.664–2.631	0.428			
Liver metastasis (yes vs. no)	1.379	0.688–2.761	0.365			
Treatment line (3 vs. ≥4)	0.486	0.257–0.919	0.026	0.550	0.288–1.051	0.071
Antiangiogenic therapy						
Anlotinib vs. endostatin	1.377	0.771–2.459	0.280			
Anlotinib vs. bevacizumab	0.710	0.304–1.657	0.428			
Bevacizumab vs. endostatin	2.127	0.857–5.280	0.104			

Abbreviations: CI, confidence interval; HR, hazard ratio; SCC, squamous cell carcinoma.

**TABLE 4:** Univariable and multivariable Cox regression analyses of factors associated with overall survival.

Characteristics	Univariable analysis			Multivariable analysis		
	HR	95% CI	*p*‐value	HR	95% CI	*p*‐value
Age (<65 vs. ≥65 years)	0.746	0.433–1.287	0.292			
Gender (female vs. male)	1.202	0.666–2.169	0.542			
Smoking history (ever vs. never)	2.831	1.442–5.559	0.003	2.491	1.244–4.988	0.010
Histology (adenocarcinoma vs. SCC)	1.156	0.880–1.519	0.298			
Brain metastasis (yes vs. no)	1.463	0.732–2.921	0.282			
Liver metastasis (yes vs. no)	1.948	0.947–4.005	0.070			
Treatment line (3 vs. ≥4)	0.488	0.256–0.930	0.029	0.621	0.320–1.205	0.159
Antiangiogenic therapy						
Anlotinib vs. endostatin	1.697	0.945–3.047	0.077			
Anlotinib vs. bevacizumab	1.427	0.584–3.488	0.436			
Bevacizumab vs. endostatin	1.041	0.408–2.653	0.934			

Abbreviations: CI, confidence interval; HR, hazard ratio; SCC, squamous cell carcinoma.

After combination therapy, 39 (66.1%) patients had ≥1 reduction in ECOG PS, including 29 (49.2%) patients with ECOG PS 1 and 10 (16.9%) with ECOG PS 0. Two of 11 patients with brain metastasis and 3 of 10 patients with liver metastasis had ≥1 reduction in ECOG PS. A statistically significant change in ECOG PS was observed after treatment (*p* < 0.05; Table 5). By the end of follow‐up, 8 (13.6%) patients had disease progression, including three patients with brain metastasis and two with liver metastasis. Of these eight patients, five still had ECOG PS 2 after progression, while the ECOG PS increased to three in three patients. For three patients with brain metastasis, the ECOG PS increased to three in one patient after progression, 1 patient’s ECOG PS increased to 3. For two patients with liver metastasis, ECOG PS was unchanged after progression (Table 6).

**TABLE 5:** Change in ECOG PS before and after treatment.

ECOG PS	Total (*n* = 59)		Brain metastasis (*n* = 11)		Liver metastasis (*n* = 10)	
	Before treatment	After treatment	Before treatment	After treatment	Before treatment	After treatment
0	0	10	0	2	0	3
1	0	29	0	4	0	2
2	59	20	11	5	10	5

Abbreviation: ECOG PS, Eastern Cooperative Oncology Group performance status.

**TABLE 6:** Change in ECOG PS for patients with disease progression during the treatment period.

ECOG PS	Total (*n* = 8)		Brain metastasis (*n* = 3)		Liver metastasis (*n* = 2)	
	Before treatment	After progression	Before treatment	After progression	Before treatment	After progression
0	0	0	0	0	0	0
1	0	0	0	0	0	0
2	8	5	3	2	2	2
3	0	3	0	1	0	0

Abbreviation: ECOG PS, Eastern Cooperative Oncology Group performance status.

## 4 DISCUSSION

This retrospective study evaluated the effectiveness and safety of immunotherapy combined with antiangiogenic therapy as third‐ or further‐line therapy in stage IV NSCLC patients with ECOG PS 2. The results indicated the sustained tolerability and good antitumor activity of this combination strategy. With this combination therapy, significant improvement in QOL was also observed, as reflected by the reduction in ECOG PS.

In the past decade, the introduction of ICIs has significantly improved the prognosis of patients with NSCLC. In previous CheckMate 017, CheckMate 057, KEYNOTE‐010, and OAK studies, ICI monotherapy showed an ORR of 14%–22.9%, a DCR of 56–58.6%, and a median PFS of 2.5–4.0 months.^10^, ^11^, ^12^ In our study, immunotherapy combined with antiangiogenic therapy resulted in an ORR of 35.6%, a DCR of 86.4%, and a median PFS of 5.5 months (95% CI: 3.8–7.3). These results suggest the better clinical benefits with combination therapy than with ICI monotherapy, although these indirect comparisons involve inherent bias.

Increasing evidence suggests a bidirectional relationship between the immune system and angiogenesis. Several studies have reported the outcomes of the combination therapy in NSCLC.^28^, ^29^ However, all the enrolled patients had good ECOG PS. The vast majority of ICI studies excluded patients with poor ECOG PS (≥2).^30^ ECOG PS 2 is the most significant negative prognostic factor and a predictor of poor survival and adverse events.^31^ In general, patients with ECOG PS 2 do not tolerate aggressive treatment, and require palliative nutritional support and pain relief. Nevertheless, many patients are still willing to attempt antitumor therapy. A review of the literature showed that only a few randomized controlled trials enrolled patients with ECOG PS ≥2. The CheckMate 817 trial showed manageable treatment‐related toxicity and a 3‐year OS rate of 20.5% with first‐line nivolumab plus ipilimumab in patients with metastatic NSCLC, including those with ECOG PS 2.^32^ In the CheckMate 171 trial, patients with advanced squamous NSCLC (including patients with ECOG PS 2 and the elderly population) treated with nivolumab had a median OS of 9.9 months, showing similar tolerability between patients with ECOG PS 2 and the overall population.^33^ In a retrospective analysis performed in 2020, the survival outcomes of NSCLC patients who received immunotherapy varied with pretreatment ECOG PS. The median OS was 14.7, 8.3, and 1.5 months in the ECOG PS 0–1, 2, and 3 groups, respectively (*p* < 0.001).^34^ Our findings showed that immunotherapy combined with antiangiogenic therapy was well‐tolerated, with a median PFS of 5.5 months (95% CI: 3.8–7.3) and a median OS of 10.5 months (95% CI: 11.2.‐13.6), and significantly improved the QOL of patients with ECOG PS 2. A number of phase II clinical trials have evaluated the efficacy of this combination as a potentially effective treatment option. Our results provide additional evidence for subsequent clinical trials.

Single‐agent immunotherapy is not effective in the treatment of patients with liver metastasis.^35^, ^36^, ^37^ Liver metastasis is common in NSCLC and is a negative prognostic indicator. In the present study, patients with liver metastasis had shorter median PFS (4.9 vs. 5.5 months, HR = 1.366, 95% CI: 0.639–2.920, *p* = 0.359) and median OS (9.2 vs. 11.1 months, HR = 1.848, 95% CI: 0.787–4.336, *p* = 0.064) than those without liver metastasis, although the differences were not statistically significant. This indicated that the combination therapy was safe and effective for NSCLC patients with ECOG PS 2 and liver metastasis. Previous studies have shown that the immune response is organ‐specific, and insufficient infiltration of CD8^+^ T lymphocyte in liver metastases leads to worse prognosis in NSCLC patients with liver metastasis.^36^, ^38^ Antiangiogenic drugs can increase the infiltration of CD8^+^ T lymphocyte and remodel the normal tumor microenvironment, thus increasing the antitumor efficacy of ICIs. Immunotherapy combined with antiangiogenic therapy is a good clinical treatment option for patients with liver metastasis from NSCLC.

In NSCLC, approximately 40% of patients will develop brain metastasis, which in turn leads to a poor prognosis.^39^ Studies have shown that in patients receiving anlotinib combined with immunotherapy, patients without brain metastasis have significantly longer median PFS than those with brain metastasis.^40^ Our results similarly showed that patients with ECOG PS 2 and brain metastasis had shorter median PFS (3.4 vs. 6.0 months, HR = 1.315, 95% CI: 0.621–2.785, *p* = 0.420) and median OS (6.0 vs. 10.7 months, HR = 1.450, 95% CI: 0.667–3.154, *p* = 0.274) than those without brain metastasis, but without statistically significant differences. However, significant improvement in QOL was observed, even in patients with brain metastasis who had disease progression during the treatment period. Thus, immunotherapy combined with antiangiogenic therapy may be effective for brain metastasis. Furthermore, patients with brain metastasis also showed good tolerance to this combination as a third‐ or further‐line therapy.

We analyzed the association between baseline characteristics and clinical response. Due to the complexity and heterogeneity of treatment response, the National Comprehensive Cancer Network guidelines recommend invasive methods to measure PD‐L1 expression.^41^ Most patients are reluctant to undergo the test and cannot afford the expensive cost, which contributes to the low proportion of patients with known PD‐L1 expression level. Therefore, this factor was not included in the Cox proportional hazard model. Our multivariable analysis found that the presence of smoking history was an independent risk factor for PFS (*p* = 0.011) and OS (*p* = 0.010).

In clinical practice, the selection of treatment options for patients should not only follow guidelines, but also be based on cost, drug availability, and patient willingness. We selected ICI combined with antiangiogenic drug for our patients according to the above factors. Although different antiangiogenic drugs were used in our study, the type of drug had no impact on PFS or OS. However, the sample size of our study was small and the association between drug type and survival still needs to be validated in further large‐scale clinical studies.

During treatment with immunotherapy combined with antiangiogenic therapy, most TRAEs were Grade 1 or 2, similar to previously reported results.^29^, ^42^, ^43^ No patients discontinued treatment due to TRAEs. However, the incidence of Grade 3–4 TRAEs was significantly higher than that of anti‐PD‐1/PD‐L1 monotherapy in previous studies (25% vs. 7–10%).^8^, ^9^, ^44^ Nevertheless, most TRAEs did not affect treatment and could be resolved. Therefore, the safety of this combination as third‐ or further‐line therapy for stage IV NSCLC patients with ECOG PS 2 was acceptable.

The limitations of the present study should be acknowledged. First, this was a single‐center retrospective study with a limited sample size, which existed unavoidable selection bias. Second, no control group was included in our study to determine the role of combination therapy by comparing it with ICI monotherapy. Large clinical trials are required to evaluate the impact of immunotherapy combined with antiangiogenic therapy as third‐ or further‐line therapy in NSCLC patients with poor ECOG PS. However, given the limited number of published prospective clinical studies in this population, our findings may still be considered relevant.

In conclusion, our findings suggest that the combination of immunotherapy and antiangiogenic therapy as third‐ or further‐line therapy shows good antitumor activity and manageable toxicity in stage IV NSCLC patients with ECOG PS 2. This combination therapy improves the physical condition of patients and contributes to improve QOL. A large prospective study is needed to further confirm our findings.

## AUTHOR CONTRIBUTIONS

**Shuo Li:** Conceptualization (equal); data curation (equal); formal analysis (equal); funding acquisition (equal); investigation (equal); methodology (equal); project administration (equal); resources (equal); software (equal); validation (equal); visualization (equal); writing – original draft (equal); writing – review and editing (equal). **Ze‐Shun Yu:** Software (equal). **Hong‐Zhi Liu:** Data curation (equal). **Shu‐Jing Li:** Data curation (equal). **Ming‐Yue Wang:** Data curation (equal). **Fang‐Ling Ning:** Conceptualization (equal); methodology (equal); supervision (lead); validation (equal); writing – review and editing (equal). **Li‐Jun Tian:** Conceptualization (equal); methodology (equal); supervision (equal); validation (equal); writing – review and editing (equal).

## FUNDING INFORMATION

This work was supported by the research fund of the Beijing Science and Technology Innovation Medical Development Foundation (KC2021‐JX‐0186‐20).

## CONFLICT OF INTEREST STATEMENT

The authors declare no conflict of interest.

## INSTITUTIONAL REVIEW BOARD STATEMENT

The study was conducted in accordance with the Declaration of Helsinki and approved by the Institutional Review Board of the Binzhou Medical University Hospital.

## CONSENT

Informed consent was obtained from all patients.

## Data Availability

The datasets generated/analyzed during the current study are available and will be provided upon request.
